# Management of retinitis pigmentosa by Wharton’s jelly derived mesenchymal stem cells: preliminary clinical results

**DOI:** 10.1186/s13287-020-1549-6

**Published:** 2020-01-13

**Authors:** Emin ÖZMERT, Umut ARSLAN

**Affiliations:** 1 Faculty of Medicine Department of Ophthalmology, Ankara University, Ankara, Turkey; 2 Ankara University Technopolis, Neorama Ofis 55-56 Yaşam Cad, No 13/A Beştepe /Yenimahalle, Ankara, Turkey

**Keywords:** Retinitis pigmentosa, Stem cell, Wharton’s jelly derived mesenchymal stem cell, Umbilical cord stem cell, Subtenon space

## Abstract

**Purpose:**

The aim of this study is to determine if umbilical cord Wharton’s jelly derived mesenchymal stem cells implanted in sub-tenon space have beneficial effects on visual functions in retinitis pigmentosa patients by reactivating the degenerated photoreceptors in dormant phase.

**Material and methods:**

This prospective, open-label, phase-3 clinical trial was conducted between April of 2019 and October of 2019 at Ankara University Faculty of Medicine, Department of Ophthalmology. 32 RP patients (34 eyes) were included in the study. The patients were followed for 6 months after the Wharton’s jelly derived mesenchymal stem cell administration, and evaluated with consecutive examinations. All patients underwent a complete routine ophthalmic examination, and best corrected visual acuity, optical coherens tomography angiography, visual field, multifocal and full-field electroretinography were performed. The quantitative results were obtained from a comparison of the pre-injection and final examination (6th month) values.

**Results:**

The mean best corrected visual acuity was 70.5 letters prior to Wharton’s jelly derived mesenchymal stem cell application and 80.6 letters at the 6th month (*p* = 0.01). The mean visual field median deviation value was 27.3 dB before the treatment and 24.7 dB at the 6th month (*p* = 0.01). The mean outer retinal thickness was 100.3 μm before the treatment and 119.1 μm at 6th month (*p* = 0.01). In the multifocal electroretinography results, P1 amplitudes improved in ring1 from 24.8 to 39.8 nv/deg2 (*p* = 0.01), in ring2 from 6.8 to 13.6 nv/deg2 (*p* = 0.01), and in ring3 from 3.1 to 5.7 nv/deg2 (*p* = 0.02). P1 implicit times improved in ring1 from 44.2 to 32.4 ms (*p* = 0.01), in ring2 from 45.2 to 33.2 ms (*p* = 0.02), and in ring3 from 41.9 to 32.4 ms (*p* = 0.01). The mean amplitude improved in 16 Tds from 2.4 to 5.0 nv/deg2 (*p* = 0.01) and in 32 Tds from 2.4 to 4.8 nv/deg2 (*p* = 0.01) in the full-field flicker electroretinography results. Full field flicker electroretinography mean implicit time also improved in 16 Tds from 43.3 to 37.9 ms (*p* = 0.01). No ocular or systemic adverse events related to the two types of surgical methods and/or Wharton’s jelly derived mesenchymal stem cells itself were observed during the follow-up period.

**Conclusion:**

RP is a genetic disorder that can result in blindness with outer retinal degeneration. Regardless of the type of genetic mutation, sub-tenon Wharton’s jelly derived mesenchymal stem cell administration appears to be an effective and safe option. There are no serious adverse events or ophthalmic / systemic side effects for 6 months follow-up. Although the long-term adverse effects are still unknown, as an extraocular approach, subtenon implantation of the stem cells seems to be a reasonable way to avoid the devastating side effects of intravitreal/submacular injection. Further studies that include long-term follow-up are needed to determine the duration of efficacy and the frequency of application.

**Trial registration:**

SHGM56733164. Redistered 28 January 2019 https://shgm.saglik.gov.tr/organ-ve-doku-nakli-koordinatorlugu/56733164/203 E.507.

## Background

The retinal pigment epithelium (RPE) forms the outer blood-retinal barrier between photoreceptor cells and choroidal blood vessels. Photoreceptor cells are vitally and functionally dependent on the RPE. The conversion of blood glucose to ATP, synthesis of proteins in the visual cycle and removal of metabolic waste takes place in the RPE. For these important processes, various peptide growth factors and their receptors are synthesized in the RPE [1–4]. More than 260 genes in the RPE are responsible for the production of these peptide fragments [5–7]. Mutations in any of these genes as well as ischemic, physical or chemical RPE damage causes retinal degeneration. Retinal degeneration may be inherited, such as in retinitis pigmentosa (RP), Stargardt’s disease, choroideremia, Best vitelliform dystrophy and Bietti’s crystalline dystrophy [8, 9]. Retinal degeneration may also be acquired through genetic mechanisms, such as age-releated macular degeneration [10, 11]. In retinal degeneration, there is a developing loss of RPE and photoreceptors, regardless of the underlying cause.

Umbilical cord Wharton’s jelly derived mesenchymal stem cells (WJ-MSCs) have significant paracrine and immunomodulatory properties [12–18]. WJ-MSCs secrete trophic factors that stimulate RPE or secrete trophic factors that are similar to those produced by RPE [19–21]. In studies using animal models, WJ-MSCs have been found to be effective in stopping the progression of retinal degeneration and for rescuing photoreceptors in the dormant phase [22–26]. WJ-MSCs are hypoimmunogenic and have significant immunomodulatory properties. WJ-MSCs have been shown to suppress chronic inflammation and prevent apoptosis in animal models of neurodegenerative and ischemic retinal disorders [27, 28]. WJ-MSCs also stimulate progenitor cells in the retina and elicit self-repair mechanisms [29, 30].

The aim of this preliminary clinical study is to investigate the efficacy of deep sub-tenon injected WJ-MSCs as a stem cell treatment modality for the management of retinitis pigmentosa, which creates outer retinal degeneration. These functional and structural effects were investigated using microperimetry, electrophysiology and spectral domain optical coherence tomography (SD-OCT). To the best of our knowledge, this is the first prospective clinical study that utilizes a large number of RP cases, and cases that are in phase-3.

## Materials and methods

Ethics committee approval for the umbilical cord Wharton’s jelly derived mesenchymal stem cell study was obtained from the Ankara University Faculty of Medicine Clinical Research Ethics Committee (19–1293-18) and was also approved by the Review Board of the Cell, Organ and Tissue Transplantation Department within the Turkish Ministry of Health (56,733,164/203 E.507). The study was performed in accordance with the tenets of the 1964 Declaration of Helsinki. Written informed consent was obtained from the patients prior to enrollment.

This prospective, open-label clinical trial was conducted between April and October of 2019 at Ankara University Faculty of Medicine, Department of Ophthalmology. 32 RP patients (34 eyes) were included in the study. The preliminary diagnosis was based on clinical history, patients’ complaints, and fundus appearance. All patients enrolled in this study underwent a complete routine ophthalmic examination, including the best-corrected visual acuity (BCVA) measurement with the early treatment of diabetic retinopathy study (ETDRS) chart (Topcon CC 100 XP, Japan). The patients were further evaluated with optical coherence tomography angiography (OCTA) (RTVue XR “Avanti”, Optovue, Fremont, CA, USA) to confirm the diagnosis that provided a typical multimodal imaging platform. Retinal and macular functions were evaluated using the Compass 24/2 visual field test (VF) (Compass, CenterVue, Padova, Italy). Photoreceptor functions were evaluated using multifocal electroretinography (mfERG) (Retiscan, Roland Germany) and with a full-field flicker ERG device (RETeval, LKC Tech. Inc., Gaithersburg, MD, USA).

Dietary supplements were suspended in RP patients 1 month before enrolling in the study because these may interfere with visual functions.

### Subjects

The study included 34 eyes from 32 RP patients and in these patients, phase-3 clinical stem cell research was conducted.

RP patients were included in this study if they met the following criteria:

- 18 years of age or older;
- Diagnosis of any phenotypic or genotypic variation of RP, confirmed by clinical history, fundus appearance, visual field (VF), electroretinogram (ERG) and genetic mutation analysis;
- Having experienced various degrees of VF loss;
- BCVA from 50 letters to 110 letters in the ETDRS chart testing (Topcon CC-100 XP, Japan);
- Mean deviation (MD) values ranging between − 33.0 and − 5.0 dB with Compass visual field analysis (threshold 24–2, Sita Standard, Stimulus 3-white);
- Intraocular pressure (IOP) of < 22 mmHg.

RP patients were excluded from the study if any of the following were found:

- The presence of cataracts or other media opacity that might affect the VF, MD, or ERG recordings;
- The presence of glaucoma, which causes visual field and optic disc changes;
- The presence of any systemic disorder (e.g.,diabetes, neurological disease, or uncontrolled systemic hypertension) that may affect visual function;
- The habit of smoking.

### Umbilical cord Wharton’s jelly derived mesenchymal stem cell preparation

**Fig. 1:**
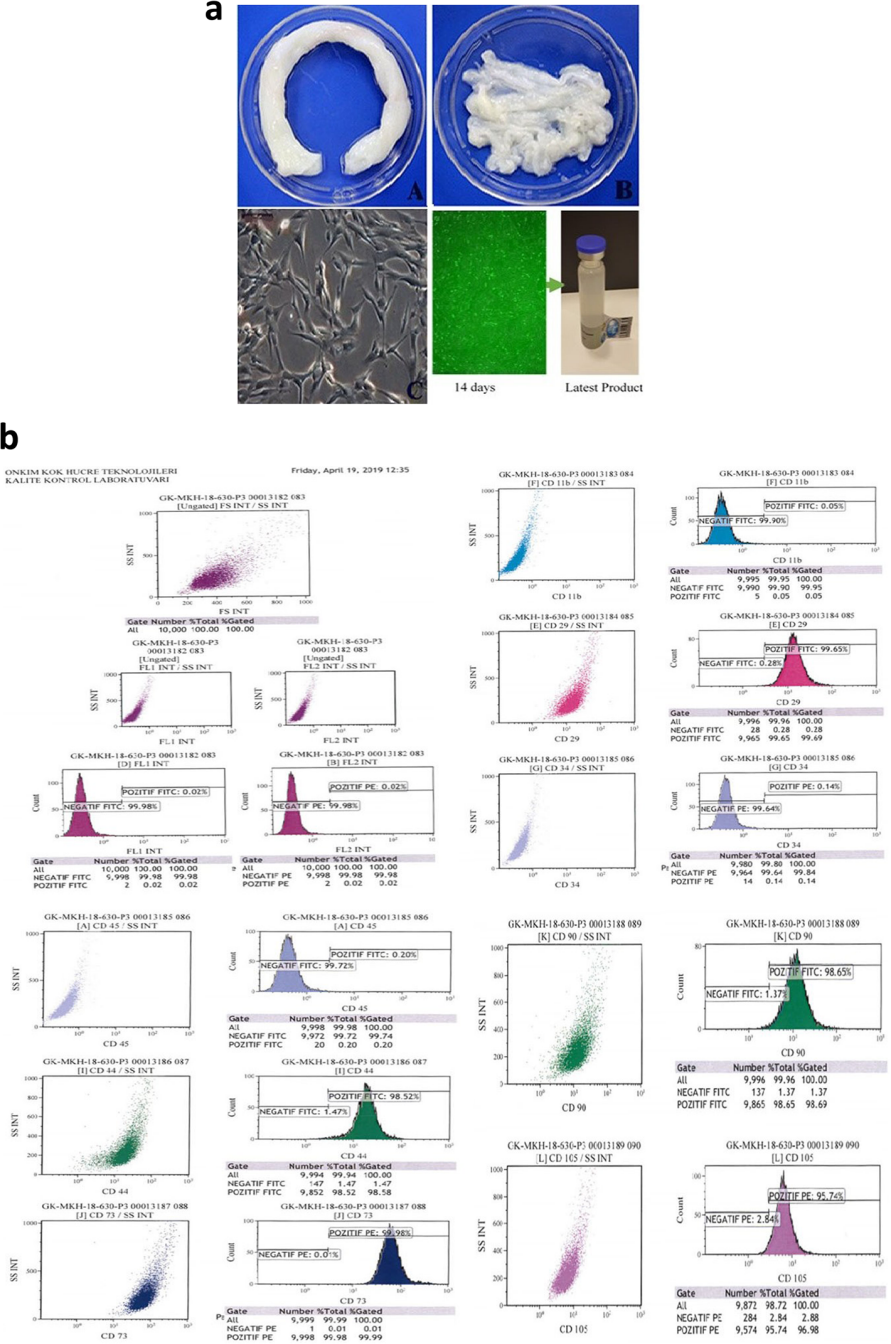
**a** Obtaining mesenchymal stem cells from umbilical cord Wharton’s jelly, morphological appearance and final injectable product. **b** Flow cytometric analysis of WJ-MSC

The mesenchymal cells that were used in this study were isolated from Wharton’s jelly of the umbilical cord that was collected allogenicly from a single donor with the mother’s consent. The umbilical cord sample was treated as follows: briefly, cord tissue was washed twice with PBS (Lonza, Switzerland) and the Wharton’s jelly part was minced using forceps and a scalpel. Minced pieces were cultivated in a cell culture dish (Greiner Bio-One, Germany) with Dulbecco’s modified Eagle’s medium F12 (DMEM)-low glucose no L-Glutamine (Bilogical Industries, Israil) with 10% human AB serum (Capricorn, Germany), 1% 10.000 U/mL penicillin and 10.000 μg/mL streptomycin (Gibco,USA). All cell preparation and cultivation procedures were conducted in a current Good Manufacturing Practice (cGMP) accredited laboratory (Onkim Stem Cell Technologies, Turkey). The culture-expanded cells were cryopreserved at P3 using standard cryopreservation protocols until their use in the following experiment. The cells were characterized at the time of cryopreservation with flow cytometric analysis to determine the expression of positive surface markers CD90, CD105, CD73, CD44, CD29, and negative for CD34, CD45 and CD11b; also, using real-time polymerase chain reaction (PCR), the expression of LDHA, HLA-DR, HLA-G, BMP2, BMP4, BMP6, JAG1, ZPF42, NANOG, POU5F1, ENG, CD44, TNF, ICAM1, VIM, THY1, VCAM1, VEGFA NES, RUNX2, SMURF1 and COL1A1 genes were analyzed. Additionally, quality control analyses like mycoplasma analysis (using PCR), endotoxin analysis (using the LAL test and sterility analysis) were also completed. Cells were solubilized from cryopreservation before being made ready for injection. Average cell viability for each treatment was over 90.0% and each patient received cell numbers between 2-6 × 10^6^ in a 1.5 ml saline solution (Fig. 1a, b).

### Injection of umbilical cord WJ-MSCs

The WJ-MSC suspension from the culture was delivered to the operating room by cold chain for use within 24 h. A total of 1.5 ml of the WJ-MSC suspension was withdrawn using a 2.5 cc syringe and was immediately injected into the subtenon space of each eye. The injection of the WJ-MSC suspensions were carried out by two ophthalmologist (EÖ - UA) using two distinct methods. Procedures were conducted under topical anesthesia with proparacaine hydrochloride drops (Alcaine, Alcon, USA) and sterile conditions. In the first method, the preplaced suture technique, a small cut was made through the conjunctiva and tenon capsule up to the sclera in the infero-nasal quadrant, 13 mm away from the limbus, for the insertion of a 20 G subtenon curved canulla (BD, Visitec, UK). Subsequently, a 7/0 vicryl suture was passed through the conjunctiva and tenon and tied down with a loop creation. A curved subtenon canulla attached to the 2.5 cc syringe filled with 1.5 ml fluid containing stem cells was inserted through the cut, and forwarded into the extraocular muscle conus until reaching the sclera. 1.5 ml of fluid was then injected. While the canulla was drawn back, a loop was tightened in order to prevent leakage. The second ophthalmologist performed a subtenon injection using a 25-gauge sharp-tip syringe without any incision into the supero-temporal region because the largest quadrant for the effective delivery of the 1.5 ml fluid containing stem cells. Both methods were used in an equal number of eyes (17 eyes for each method). In both methods, in order to expose the more sub-tenon space in the chosen region, a traction by a 5/0 atraumatic silk suture with a round needle was exerted into the limbus, pulling away from the cut / injection site. In both methods, it was confirmed using orbital ultrasound (Quantel, Cournon d’Auvergne, France) that the injection was delivered to the deep sub-tenon region near the sclera and within the extraocular muscle conus. Postoperatively, loteprednol + tobramycin combination eye drops were given 4 times per day for 1 week and oral amoxicillin clavulonate was given at 1 g, twice a day for 5 days.

The patients were followed for 6 months after the WJ-MSC injection and underwent 5 consecutive examinations to monitor the individuals closely and record any possible adverse/side effects. The quantitative results were obtained by comparing the pre-injection and final examination (6th month) values. The primary aim of this clinical study was to assess the effects of WJ-MSCs on BCVA, VF, outer retinal thickness (ORT), mfERG and full-field flicker ERG. The secondary aim of the study was to investigate whether both surgical techniques are safe and the amount of stem cells used is sufficient to elicit clinical responses.

For VF analysis, in order to avoid mistakes during the test, practice rounds were carried out three times before the WJ-MSC injection of each eye. These visual field practice tests were completed using the same parameters as the real test to exclude learning effects.

To evaluate retinal functions, mfERG could be performed on patients who had sufficient fixation according to the ISCEV standard protocol [31–33]. The mfERG measures neuroretinal function (postreceptoral responses, cone mediated ON and OFF bipolar cells, and inner retinal cell contributions) in localized retinal areas. The amplitude (nv/deg2) and implicit times (ms) of the first-order kernel mfERG responses (N1 and P1 waves) were obtained and grouped into five rings (ring 1, central 2°; ring 2, 2–5°; ring 3, 5–10°; ring 4, 10–15°; ring 5,> 15°). In all subjects, the mfERG testing protocol was began 20 min after preadaptation to an ambiently lit environment equivalent to the mean luminance of the stimulus at 100 cd/m^2^. Pupils were pharmacologically (with tropicamide 1%) dilated to 8–9 mm. The cornea was anesthetized with proparacaine hydrochloride drops. The mfERGs were recorded monocularly, patching the contralateral eye using a DTL electrode. A small gold skin ground electrode was placed at the center of the forehead after preparing the skin with abrasive gel. Meanwhile, a skin electrode was placed at the outer canthus to be used as a reference. mfERG was performed by correcting refraction errors. The multifocal stimulus, which consisted of 61 scaled hexagons, was displayed on a high resolution, black and white cathode ray tube (CRT) monitor with a frame rate of 75 Hz. The signal was amplified (gain 100,000) and filtered (band pass 3–300 Hz). After automatic rejection of artifacts, the first-order kernel response, K1, was examined. These parameters were obtained from five concentric annular retinal regions (rings) centered on the fovea.

Full-field flicker ERG is a noninvasive objective test that measures the electrical activity of the retina in response to a light stimulus. The 30 Hz flicker ERG reveals a response from the cone bipolar cells. Flicker stimulation is valuable for studying the neurovascular coupling, which is a physiological process, that adjusts the microcirculation in response to neural activity [34, 35]. Full-field flicker ERGs were recorded without mydriasis using the RETeval system. The measurements were taken according to the instructions provided with the instrument for both eyes. We used the 16 and 32 Tds protocol, which combines implicit time and amplitude to create a numerical result.

### Time frame

The patients were checked during the following time points:

- Before application: a period of 3 months prior to the WJ-MSC application
- 0 (baseline): just before the WJ-MSC injection
- 1: 1st month after injection
- 2: 2nd month after injection
- 3: 3rd month after injection
- 4: 6th month after injection

### Primary outcome measure

- ETDRS visual acuity (time frame: 0, 1, 2, 3 and 4)

Visual acuity was measured at the 0,1,2,3 and 4 time points. The visual acuity scores obtained from the baseline testing and the final examination were analyzed and compared (using statistical tests) to determine effectiveness.

### Secondary outcome measures

- Visual field sensitivity (time frame: before application, 0, 1, 2, 3 and 4)

A Compass visual field analyzer, threshold 24–2 modality, was used at the 0, 1, 2, 3 and 4 time-points. In addition, it was used three times before application during experimentation to exclude the learning effect. The MD values, which were obtained from the baseline test and the final examination, were analyzed and compared (using statistical tests) to determine the effectiveness of the treatment.

- Outer retinal thickness (time frame: before application, 0, 1, 2, 3 and 4)

The structural parameters were measured on OCTA at the 0, 1, 2, 3 and 4 time points. Outer retinal thickness (ORT): This is the thickness from the outer plexiform layer to the Bruch membrane in the 3 × 3 mm area of the fovea measured (and recorded automatically) by the multimodal imaging OCTA device.

- Amplitudes of multifocal electroretinogram [time frame: 0 and 4]

The retinal electrical responses from mfERG were measured in patients by correcting refraction errors at the 0 and 4 time points. The amplitudes of each ring obtained during baseline testing and in the final examination were analyzed and compared (using statistical tests) to determine the effectiveness of the treatment.

- Implicit times of multifocal electroretinogram (time frame: 0 and 4)

The implicit times of each ring obtained from the baseline testing and the final examination were analyzed and compared (using statistical tests) to determine the effectiveness of the treatment. Full-field flicker electroretinogram (time frame: 0, 1, 2, 3 and 4).

The amplitudes and implict times obtained from the baseline testing and the final examination were analyzed and compared (using statistical tests) to determine the effectiveness of the treatment. mfERG was started as soon as necessary permissions were obtained due to electrophysiology laboratory density. Some deviations in the time frame were found to not change the mfERG results.

### Definition of safety outcome

Intraocular/intraorbital inflammation, proptosis, diplopia, afferent pupillary defect, corneal/lenticular haze, ocular allergic reactions, intravitreal/subretinal/macular hemorrhages, vitreoretinal interface alterations, retinal tear(s)/retinal detachment (exudative, rhegmatogenous), intraocular pressure change from baseline (≤5 mmHg) were considered to be serious adverse ocular events. Besides the routine ophthalmic examinations, OCTA multimodal imaging was also used to detect and confirm the presence of complications and anatomical changes during each examination for the study period. Systemic allergic reactions and anaphylaxis were considered to be systemic side effects.

### Statistical methods

The statistical comparisons were made primarily between the baseline and final values from the same eye. The BCVA and parametric results for visual field, ORT, mfERG and full field flicker ERG were analyzed using a Student’s paired t-test. Results are presented as means and standard deviations. *P* values less than 0.05 are considered statistically significant. A 95% confidence interval for the difference in means was used for double confirmation. Analyses were carried out with SPSS for Windows (v22; IBM Corp.; Armonk, NY, USA).

## Results

Thirty four eyes from 32 RP patients of various genotypes that were enrolled in phase-3 clinical stem cell research were included in the study. Of the 32 patients, 18 were male and 14 were female; their median age was 38.7 years (range, 18–58 years).

BCVAs, visual field MD values, and outer retinal thickness values just before the stem cell injection and at 6 months post-injection are displayed in Table 1. The statistical analyses of the these parameters are presented in Table 2, which were determined to all be statistically significant (*p* = 0.01). The mean BCVA was 70.5 letters prior to stem cell treatment and 80.6 letters 6 months post-treatment (*p* = 0.01).

**Table 1:** Changes in BCVA, visual field MD and ORT values after WJ-MSC applications in 34 eyes (32 patients)

No	Eye	BCVA		Visual field MD		Outer Retinal Thickness	
		Before	After	Before	After	Before	After
1	R^i^	100	110	28,3	27,7	85.0	152.2
2	L^i^	80	87	28,0	26,6	93.4	120.4
3	R^c^	80	88	28,3	27,3	117.4	117.8
4	L^c^	84	88	28,2	26,7	118.2	145.4
5	L^c^	50	74	29,2	26,1	103.0	105.7
6	L^c^	80	85	28,9	25,8	129.0	131.9
7	R^c^	50	56	19.4	9,4	116.0	126.3
8	L^c^	80	87	27,6	26,4	101.4	102.2
9	L^i^	72	79	25,3	19,2	79.2	135.6
10	L^i^	50	55	18,7	14,4	105.0	111.8
11	R^c^	65	77	29,2	28,1	92.2	128.7
12	R^i^	65	74	29,6	28,8	128.2	145.6
13	L^i^	65	87	28,0	25,7	101.0	104.8
14	R^c^	50	69	19,5	5,7	100.4	110.0
15	L^i^	89	110	28,4	16,4	84.8	120.0
16	R^i^	80	83	28,9	28,2	128.6	98.4
17	L^c^	50	59	29,1	29,0	105.6	122.8
18	L^c^	80	85	28,6	27,3	89.0	94.8
19	R^i^	50	65	26,1	23,9	82.6	109.0
20	R^c^	60	77	13,9	13,3	84.2	86.8
21	R^c^	50	59	29,9	29,4	85.2	97.0
22	L^i^	77	87	28,6	27,5	86.0	116.0
23	R^i^	70	70	25,5	25,6	91.0	91.6
24	L^i^	98	110	26,1	25,9	118.2	152.4
25	L^c^	85	91	29,1	28,9	106.0	132.8
26	L^c^	89	110	25,9	23,2	109.3	122.0
27	R^i^	85	94	28,8	28,7	120.2	135.8
28	R^i^	50	55	28,5	27,9	116.0	122.8
29	R^c^	50	50	29,4	29,4	117.0	117.0
30	L^i^	89	94	27,4	25,2	110.0	140.4
31	R^c^	74	85	28,9	27,6	101.0	106.0
	L^i^	80	87	28,6	28,1	96.9	101.3
32	R^c^	60	77	29,4	29,4	70.4	111.8
	L^i^	60	77	28,1	27,7	72.6	112.4

*BCVA* best-corrected visual acuity, *ETDRS* early treatment of diabetic retinopathy study charts (letters point);

*MD* mean deviation (dB)

Outer retinal thickness (μm)

i: injection by 25 G sharp-tip needle

c: application by subtenon curved canulla with preplaced suture

**Table 2:** Comparison of BCVA, Visual field MD and Outer Retinal Thickness values at baseline and final examination (6th month)

Parametres	Baseline	Final	p	%95 CI (L, U)
	X ± SD	X ± SD		
BCVA	70,5 ± 15,71	80,62 ± 16,26	0,01^*^	(−12,33, −7,9)
Visual field MD	27,25 ± 3,41	24,72 ± 5,98	0,01^*^	(1,05, 4,00)
Outer Retinal Thickness	100,26 ± 15,64	119,11 ± 17,07	0,01^*^	(−25,42, −13,26)

BCVA, best-corrected visual acuity; ETDRS, early treatment of diabetic retinopathy study charts (letters point);

MD, mean deviation (dB)

Outer retinal thickness (μm)

%95 CI L and U, 95% confidence interval of the difference (mean)

* Bold: Statistically signigicant

**Fig. 2:**
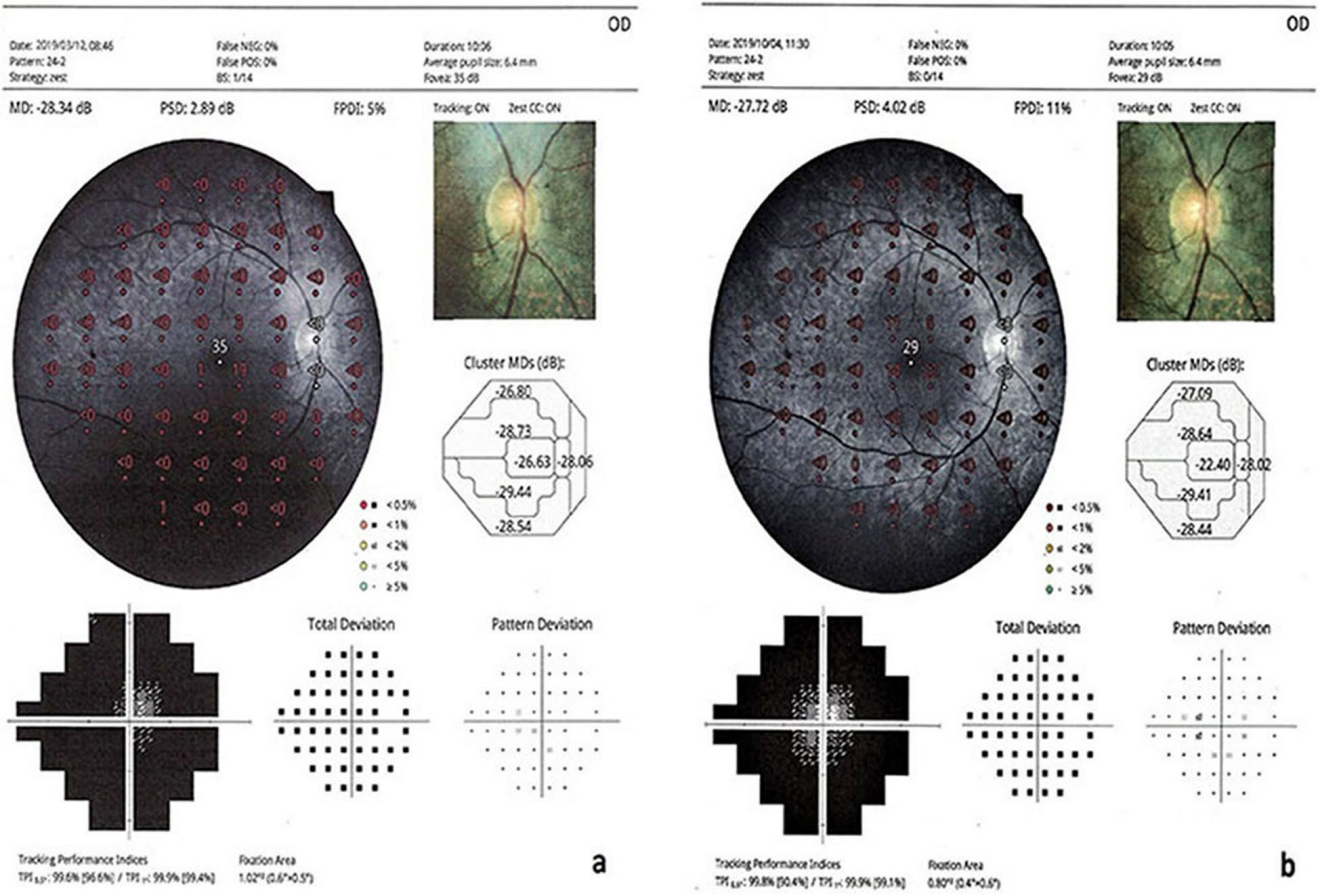
**a, b** Visual field changes in the WJ-MSC treatment (Table 1, patient no. 1: right eye). **a:** before the application, **b**: 6 months later after the application

**Fig. 3:**
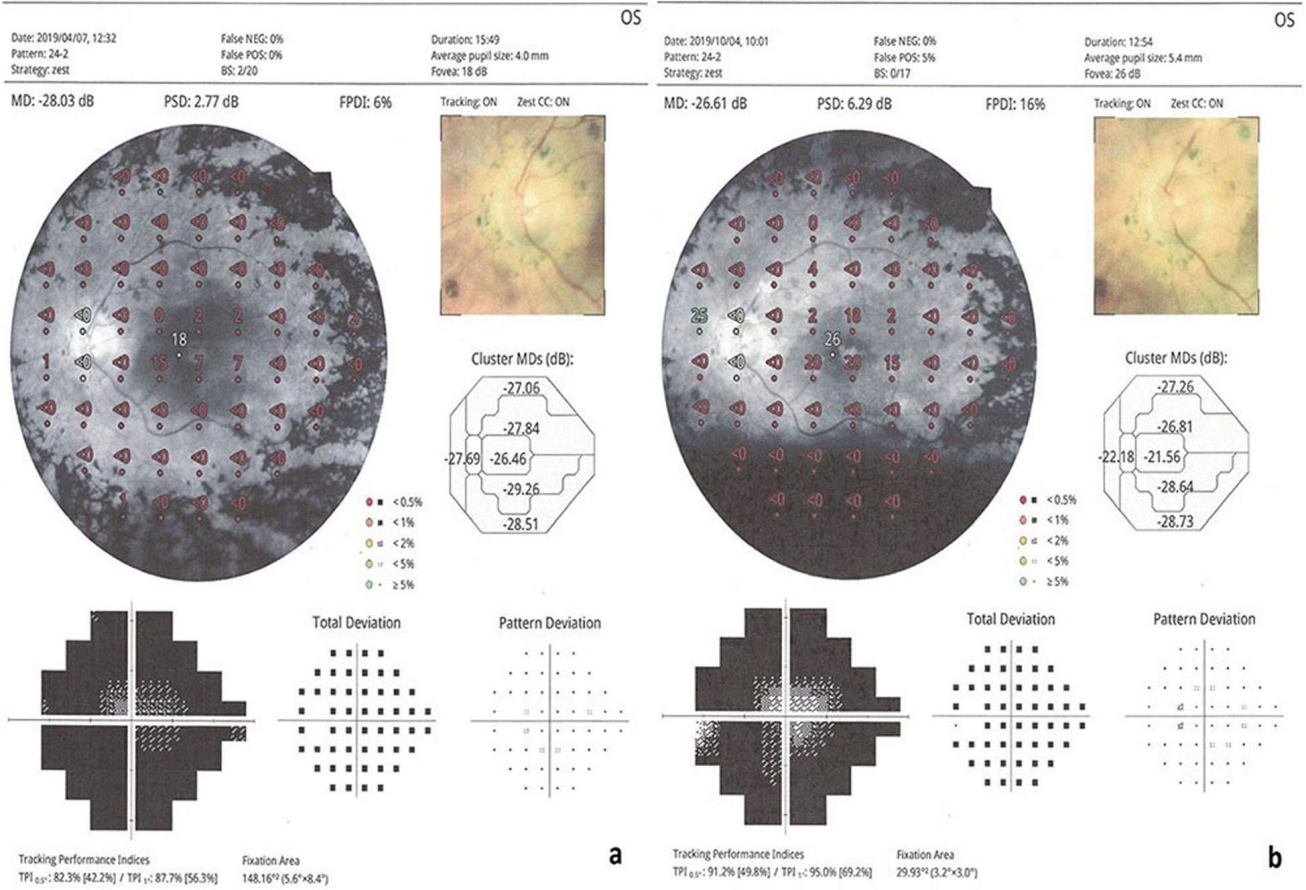
**a, b** Visual field changes in the WJ-MSC treatment (Table 1, patient no. 2: left eye). **a:** before the application, **b**: 6 months later after the application

The mean visual field MD value was 27.3 dB before WJ-MSC treatment and 24.7 dB 6 months after treatment (*p* = 0.01) (Figs. 2 and 3).

**Fig. 4:**
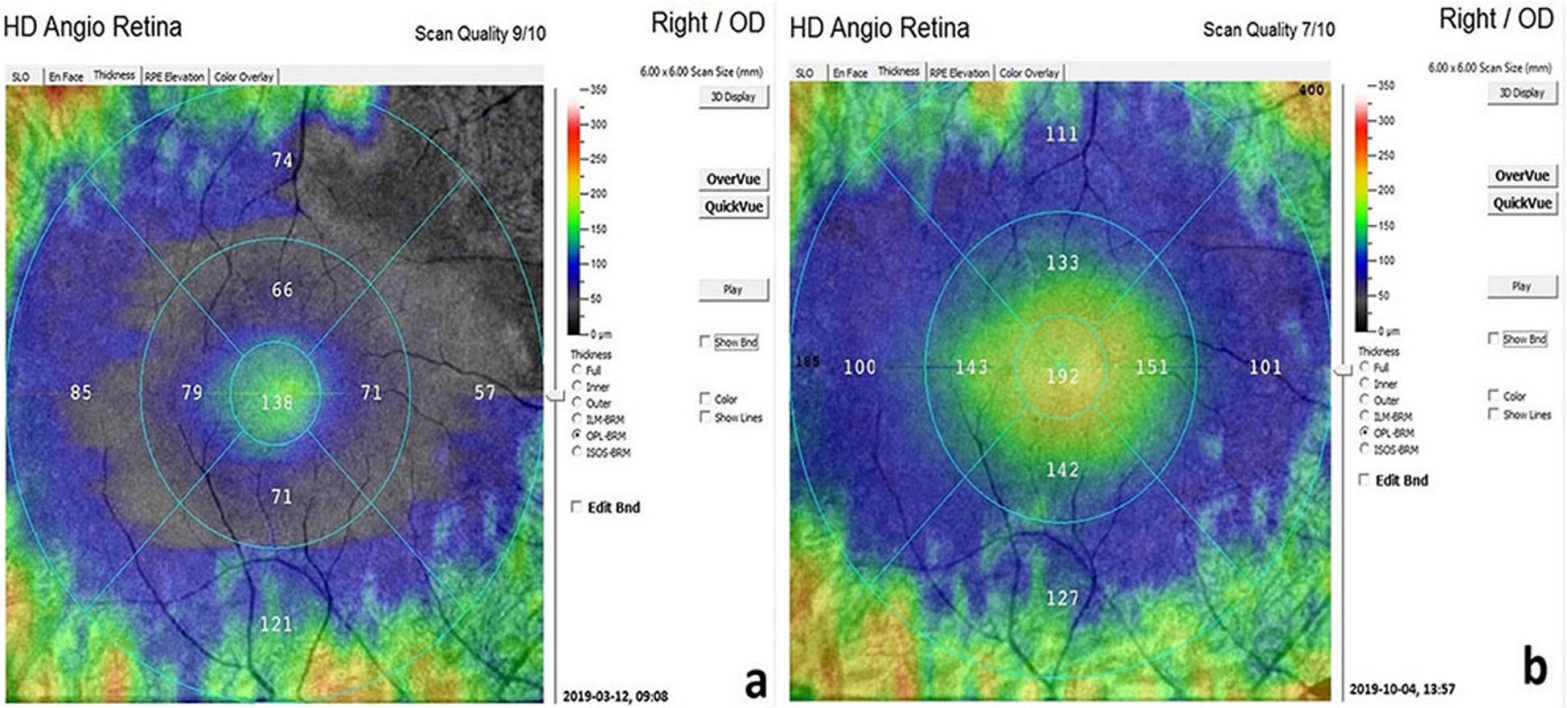
**a, b** Outer retinal thickness changes in the WJ-MSC treatment (Table 1, patient no. 1: right eye). **a**: before the application, **b**: 6 monts later after the application**.** (In order for the assessment to be meaningful, the threshold scan value should be 5 and above. Reference: OCTA device user manual)

**Fig. 5:**
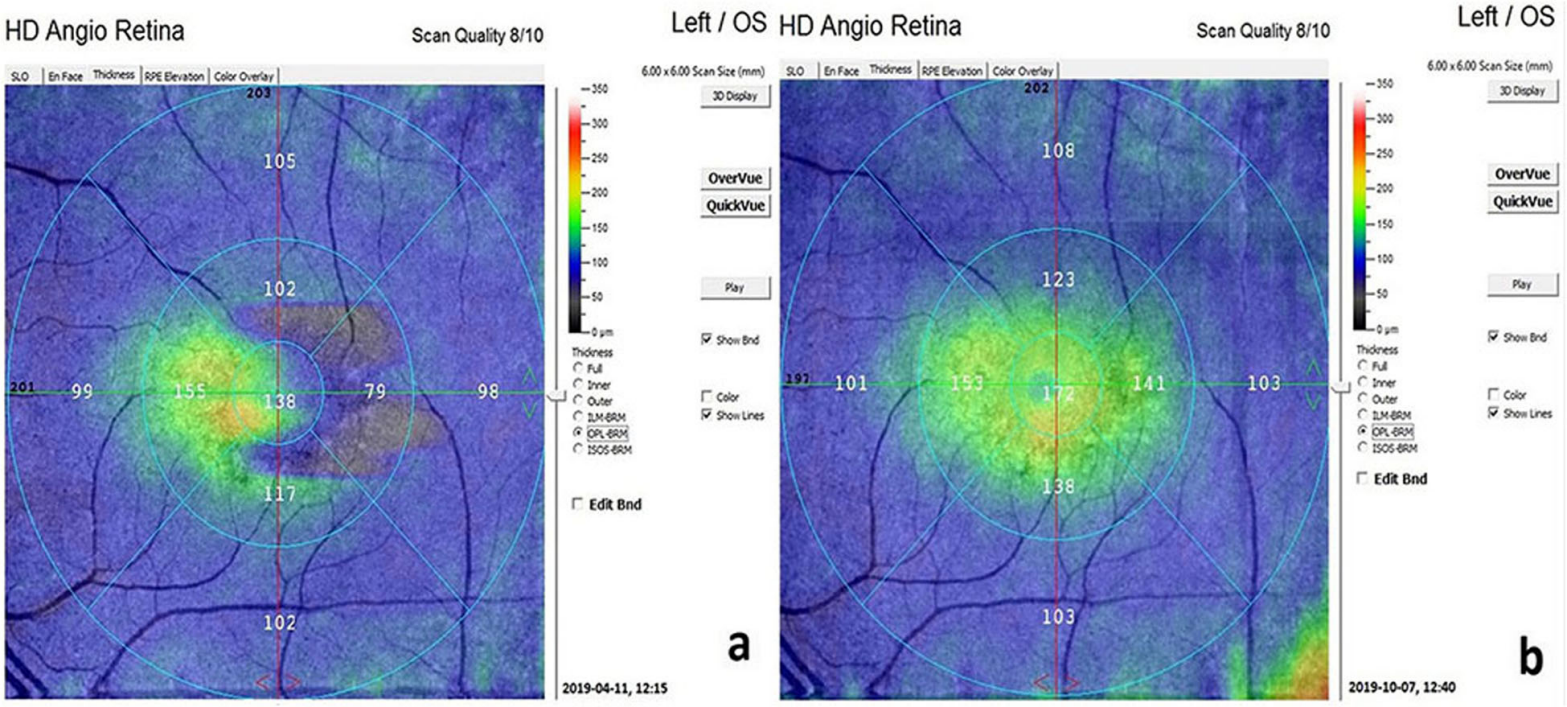
**a, b** Outer retinal thickness changes in the WJ-MSC treatment (Table 1, patient no. 4: left eye): **a:** before the application, **b**: 6 months later after the application

The mean outer retinal thickness was 100.3 μm before WJ-MSC treatment and 119.1 μm 6 months after treatment (*p* = 0.01) (Figs. 4 and 5).

Statistical results of the mfERG changes are presented in Table 3 and the full-field flicker ERG results are described in Table 4.

**Table 3:** Comparison of mfERG parameters (P1) at the baseline and final examination (6th month)

Ring	P1 amplitude (nv/deg^2^)				P1 implict time (ms)			
	Baseline	Final	*P* value	%95 CI (L, U)	Baseline	Final	*P* value	95% CI (L, U)
	X ± SD	X ± SD			X ± SD	X ± SD		
1	*24,77 ± 17,87*	*39,77 ± 21,19*	0,01*	*(−21,61,-8,38)*	*44,17 ± 10,91*	*32,36 ± 10,22*	0,01*	*(−21,61,-8,38)*
2	*6,78 ± 4,3*	*13,62 ± 9,09*	0,01*	*(−10,02,-3,65)*	*45,17 ± 8,47*	*33,22 ± 9,84*	0,02*	*(−10,02,-3,65)*
3	*3,11 ± 3,86*	*5,68 ± 4,89*	0,02*	*(−3,76,-1,38)*	*41,99 ± 10,77*	*32,4 ± 11,47*	0,01*	*(−3,76,-1,38)*
4	2,03 ± 1,37	2,74 ± 2,79	0,11	(−1,6,0,17)	47,31 ± 6,22	47,31 ± 6,22	0,26	(−1,6,0,17)
5	1,06 ± 0,84	1,13 ± 1,23	0,66	(−0,38,0,24)	48,49 ± 6,14	48,37 ± 6,68	0,47	(−0,38,0,24)

X, mean values obtained from 34 eyes (32 patients); *SD* standard deviation;

%95 CI L and U, 95% confidence interval of the difference (mean)

* Bold: Significant *p* values and related data (i.e., coefficient and standard error) are set in italics

**Table 4:** Comparison of full- field flicker ERG parameters at the baseline and final examination

Flash	Amplitude (nv/deg^2^)			Implict time (ms)		
	Baseline	Final	*P* value	Baseline	Final	*P* value
	X ± Std Err	X ± Std Err		X ± Std Err	X ± Std Err	
16 Tds	*2,37 + 1,85*	*5,03 + 4,21*	0,01*	*43,28 + 5,50*	*37,91 + 7,88*	0,01*
32 Tds	*2,44 + 2,35*	*4,81 + 3,70*	0,01*	39,48 + 9,44	36,61 + 7,47	0,11

X, mean values obtained from 34 eyes (32 patients); *SD* standard deviation;

%95 CI L and U, 95% confidence interval of the difference (mean)

*Bold: Significant *p* values and related data (i.e., coefficient and standard error) are set in italics

**Fig. 6:**
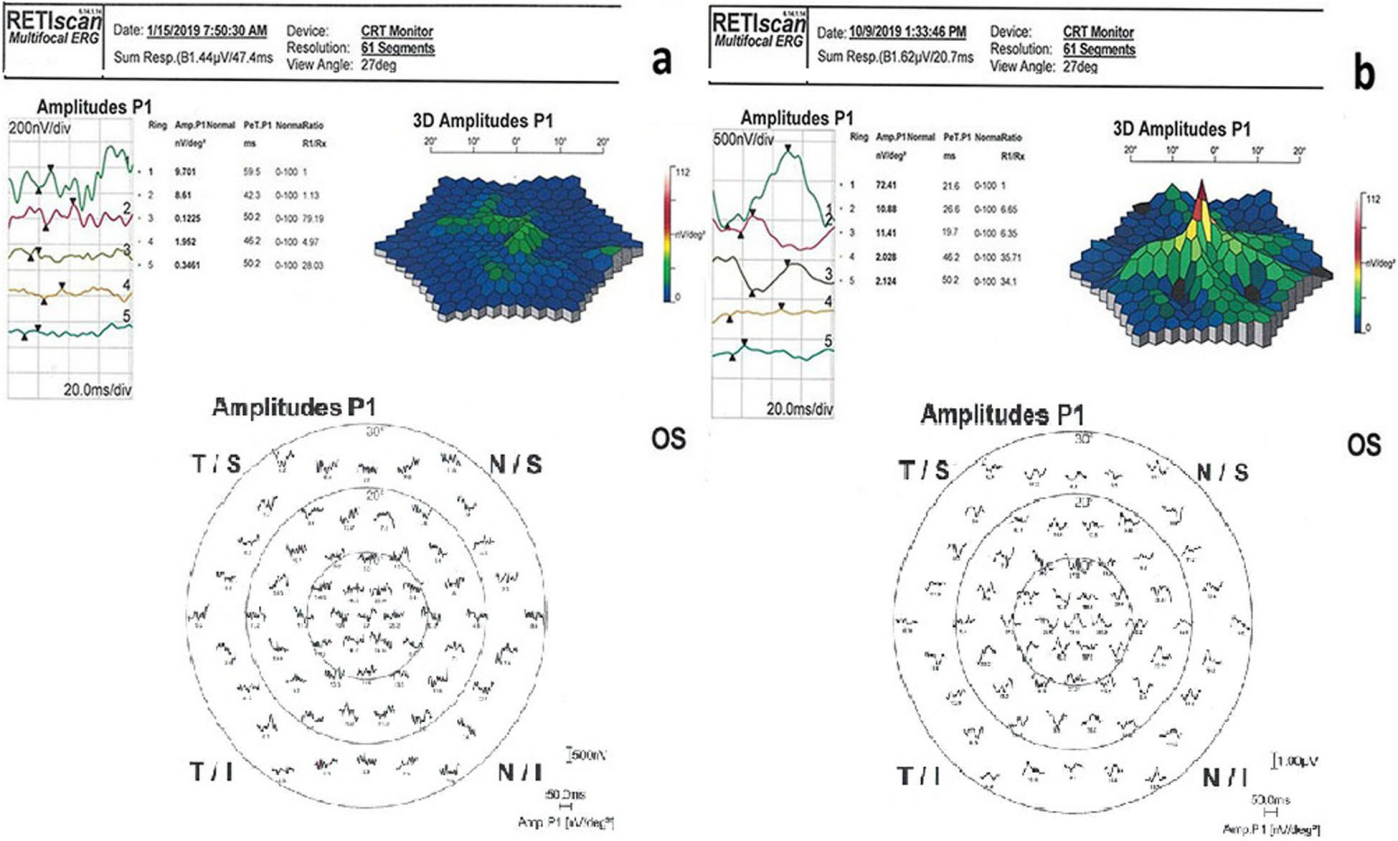
**a, b** mfERG improvement in the WJ-MSC Treatment (Table 1, patient no. 18: left eye). **a**: before the application, **b:** 6 months later after the application

**Fig. 7:**
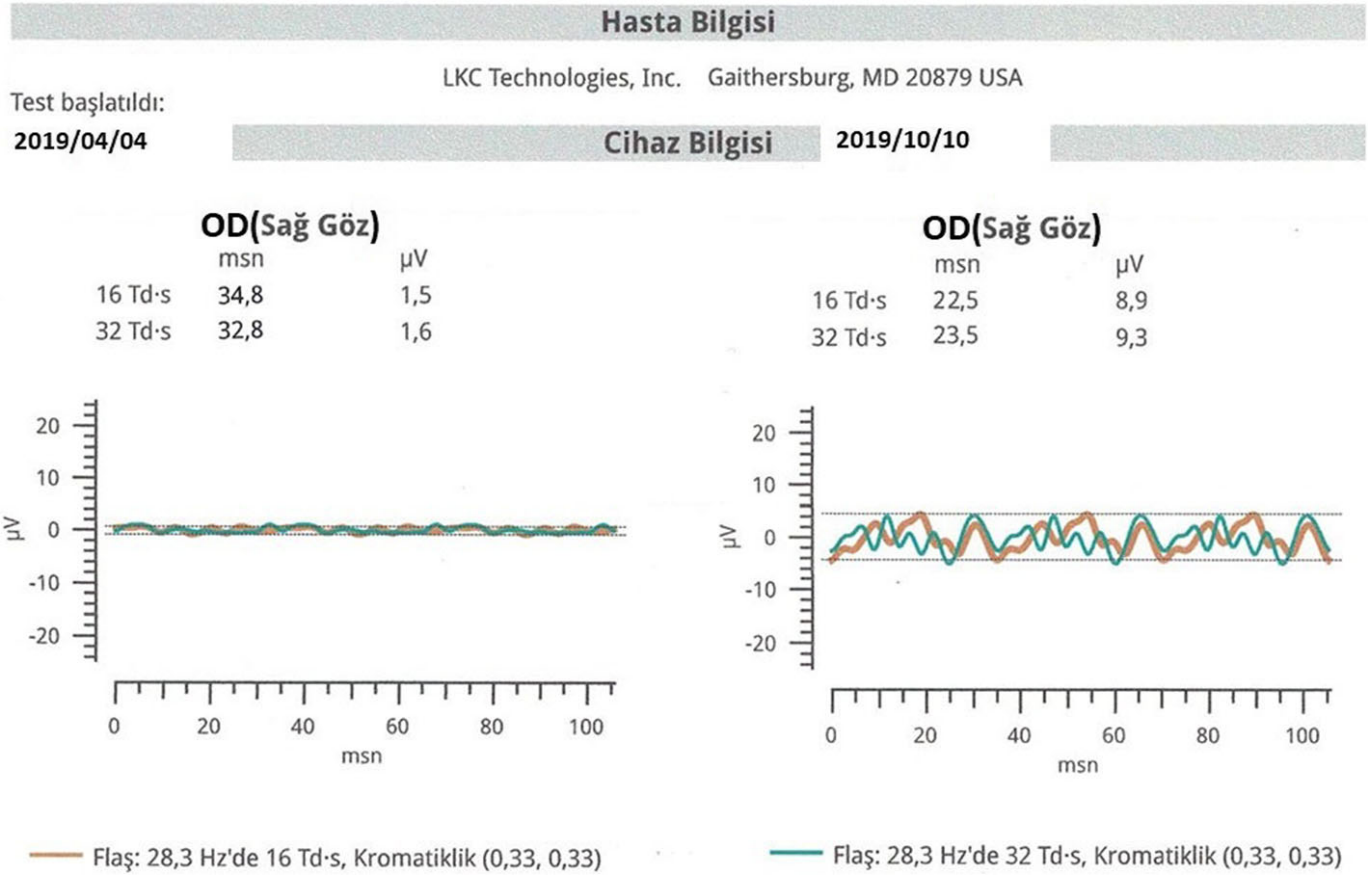
Full-field flicker ERG improvement during the follow-up (6 months) after the WJ-MSC applecation (Table 1, patient no. 11: right eye)

Regarding the mfERG results, of the 34 eyes (from 32 subjects), P1 amplitudes improved in ring 1 from 24.8 to 39.8 nv/deg2 (*p* = 0.01), in ring 2 from 6.8 to 13.6 nv/deg2 (*p* = 0.01), and in ring 3 from 3.1 to 5.7 nv/deg2 (*p* = 0.02). mfERG P1 implicit times improved in ring 1 from 44.2 to 32.4 ms (*p* = 0.01), in ring 2 from 45.2 to 33.2 ms (*p* = 0.02), and in ring 3 from 41.9 to 32.4 ms (*p* = 0.01). These changes were all found to be statistically significant. There were no significant changes in rings 4 and 5. The mean amplitude improved in 16 Tds from 2.4 to 5.0 nv/deg2 (*p* = 0.01) and in 32 Tds from 2.4 to 4.8 nv/deg2 (*p* = 0.01) in the full field flicker ERG results. These changes were also statistically significant. Full field flicker ERG mean implicit time also improved in 16 Tds from 43.3 to 37.9 ms (*p* = 0.01) (Figs. 6 and 7).

We found no statistically significant changes in any of the parameters of the untreated fellow eyes during the 6-month follow-up period. The mean BCVA was 70.6 letters at the intial exam and 71.9 letters at the last exam (*p* = 0.81). The mean visual field MD value was 27.4 dB at the initial exam and 27.1 dB at the last exam (*p* = 0.88). The mean outer retinal thickness was 102.1 μm at the begining and 104.0 μm at the last exam (*p* = 0.84).

**Fig. 8:**
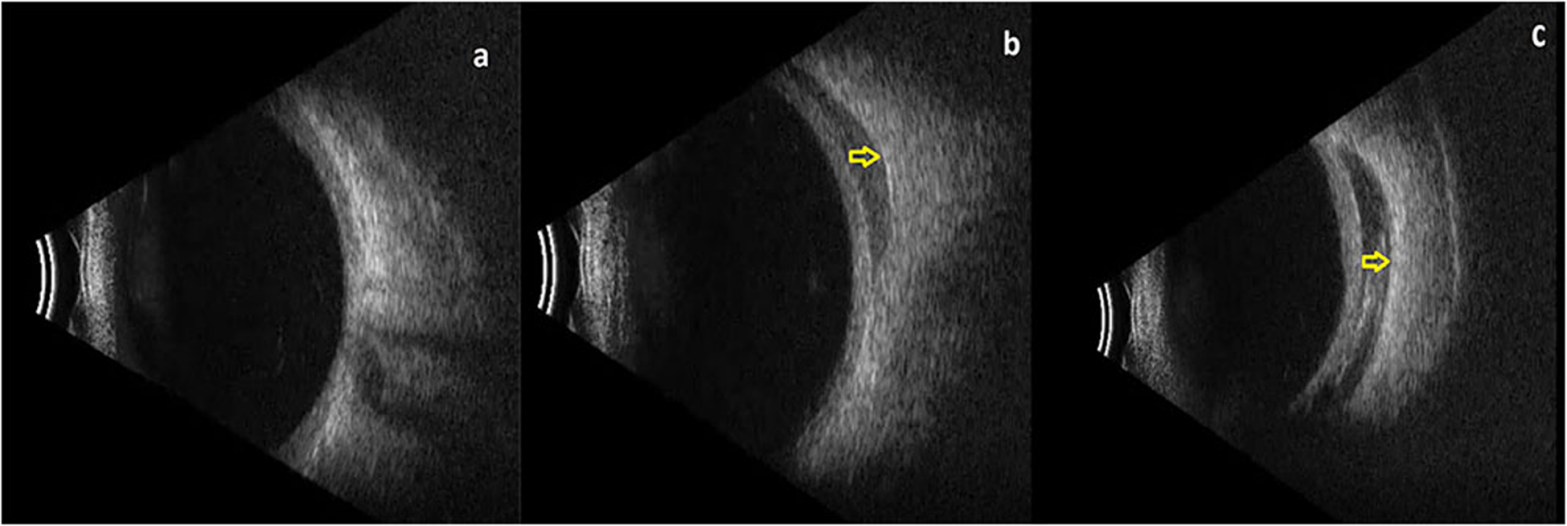
**a-c** Ultrasonographyic view of WJ-MSC implantation into the deep subretinal space within the extraocular muscle conus**; a:** before the application (Table 1, patient no. 1), **b:** injection via 25 G sharp-tip needle (Table 1, patient no. 1), **c**: placement via 20 G curved subtenon canulla with pre-placed suture to prevent the leakage (Table 1, patient no. 4)

We found no difference between the two stem-cell implantation methods in terms of the follow-up parameters. The delta change of BCVA was 11.6 letters in the injector group and 10.6 letters in the cannula group (*p* = 0.8). The delta change of the visual field MD value was 2.1 dB in the injector group and 2.5 dB in the cannula group (*p* = 0.6). The delta change of ORT was 10.7 μm in the injector group and 11.8 μm in the cannula group (*p* = 0.7). After placement of the stem cells into the sub-tenon space by either method, typical appearance on the orbital ultrasound was observed in all eyes (hyporeflective space adjacent to sclera within the muscle conus) (Fig. 8a-c). There was no issues or complications in terms of the stem-cells’ administration into the deep sub-tenon space by either method.

During the follow up period, we did not encounter any adverse effects in the studied eyes, except in one case in which the amplitute of the prexisting nistagmus increased temporarily. The research was monitored by an independent board for side effects.

## Discussion

Retinal pigment epithelium (RPE) is a single-layer barrier between the choroidal blood vessels and the sensorial retina. Photoreceptor cells are vitally and functionally dependent on RPE. The conversion of blood glucose to ATP, synthesis of proteins in the visual cycle, and removal of metabolic waste takes place in the RPE. For these important processes, various peptide growth factors and their receptors are synthesized in the RPE [1–4]. More than 260 genes in the RPE are responsible for the production of these peptide fragments. Mutations in any of these genes lead to retinitis pigmentosa [5–7]. RP is a genetic disorder with progressive photoreceptor loss and can be inherited very differently and affects between 1 in 3–8 thousand people worldwide [36, 37]. Symptoms begin with nyctalopia, persist with progressive loss of visual field, and eventually develop legal blindness [37]. Affected photoreceptors undergo apoptosis, which results in a reduced thickness of the outer nuclear layer and abnormal pigmentary deposits [38]. When the activity of growth factors decrease in the photoreceptor microenvironment, the cells first enter into sleep mode (dormant phase) and then apoptosis develops. Time from dormant phase to apoptosis differs across individual genotypes [22–26]. GFs such as neural growth factor (NGF), brain-derived neurotrophic factor (BDNF) and ciliary neurotrophic factor (CNTF) can significantly slow retinal degeneration and stop the progression in clinical and preclinical trials [38–40].

Mesenchymal stem cells (MSCs) are well known for secreting a broad range of regenerative, anti-inflammatory, anti-apoptotic and anti-fibrotic factors. These factors include NGF, BDNF, CNTF and glial-derived neurotrophic factor (GDNF) [41, 42]. These factors provide vital and functional microenvironment balance of cells but most of this microenvironment acts locally and is short-lived. MSCs produce exosomes that contain growth factors, mRNA, microRNA and mitochondrial companents. This content ensures that the exosomes are long-acting [42–44].. MSCs are nonhematopoietic multipotent stromal cells that can be isolated from different sources, including bone marrow, adipose tissue, umbilical cord tissue, cord blood, placenta, dental pulp and amniotic fluid [12, 45, 46]. Umbilical cord Wharton’s jelly derived mesenchymal stem cells (WJ-MSCs) are superior to other cell types in retinal degeneration in terms of how easily we can obtain these cells, as well as their tissue compatibility, rapid proliferation, long-term efficacy in the transplanted tissue, high paracrine effect, immunomodulation effect, non-tumoral side effect, and because these cells have similar features as the retinal pigment epithelium [13–21]. For these reasons, we chose to use WJ-MSCs in our clinical research.

For the treatment of degenerative and ischemic retinal diseases, previous preclinical and clinical studies that utilize MSC have used different routes of administration. In intravitreal and subretinal applications, severe complications have been reported which include proliferative vitreoretinal bands, tractional retinal detachment, exudative retinal detachment etc. [41, 47–51]. Suprachoroidal, subtenon or peribulbar administration methods are not reported to have serious complications [52–54]. Previous studies on mesenchymal stem cells have shown that the subtenon region acts as a natural cell culture medium by peribulbar administration. Regarding treatments that use supracoroidal adipose stem cells, macrophages are activated due to surgical trauma, and no MSC is reported in the tissue 10 months after treatment [55, 56]. GFs perform their activity by binding to the tyrosine kinase (Trk) receptors, which are commonly found around the limbus and optic nerve [57, 58]. Molecules smaller than 75 kDa can passively disperse through the sclera into the subretinal space. Molecules greater than 75 kDa can be dispersed through the sclera by changing the electrical charges using electrical/electromagnetic iontophoresis such as Magnovision™ [59–64]. Growth factors secreted by MSCs in the subretinal space activate the cells in the dormant phase and stimulate the progenitor cells (embriyonic remnants) in the retina [12–26, 29]. We preferred to use the deep sub-tenon space as a microenvironment in order to prevent the devastating adverse effects of intravitreal/subretinal injection. We also compared two routes of administration: from the infero-nasal quadrant with a 20 gauge subtenon cannula with a preplaced suture (to avoid leakage) and from a supero-temporal quadrant with a 25 gauge injector. In both methods, consistent and typical appearance was detected for all eyes using an orbital ultrasound, which suggests we achieved a proper application with both methods. We found no difference between the functional results with either method. Therefore, we think that the 25 G superotemporal subtenon injection method is appropriate and should be preferred because it does not require suture and is less traumatic, although the most appropriate preference warrants further investigation. We found no significant changes in any of the parameters in the untreated fellow eyes. This indicates that the effects of WJ-MSCs are due to spesific local transscleral receptor-mediated transport. There were no serious adverse events or ophthalmic/systemic side effects during the 6 months of follow-up. No immune rejection reactions were detected. Subtenon region is a slow release natural drug storage area. This site acts as both a natural culture medium for WJ-MSC and an immune protection site. The avascularity of the natural space between the sclera and the conjunctiva ensures that this region is relatively immune-protected [65]. The ability of the growth factors secreted by the stem cells to pass through the subretinal space are the ideal properties for this region to function as the site of stem cell application. The WJ-MSCs do not synthesize major histocompatibility complex (MHC) class II antigen, which do not produce immune rejection reactions [66].. Both the site of administration and the immune properties of the cell, although allogeneic, reduce (considerably) the likelihood of a rejection reaction in practice.

In our study, we observed that the increases in BCVA and visual field MD values were correlated with outer retinal thickness at each control. mfERG results showed significant improvement in P1 amplitudes and implict time values in the first, second and third rings. These data confirm that the cells are implanted after WJ-MSCs application, and that the paracrine effects are increased (progressively) after the implantation process. Cells passing into the dormant phase make their stoplasms more solid. The stoplasms of the reactivated cells become more liquid [67].. The growth factors secreted by the WJ-MSCs may lead to reactivation of the photoreceptors in the dormant phase and the regeneration of synaptic connections [68, 69]. A significant increase in the outer retinal thickness and improvement in visual functions can be explained through this mechanism.

Significant improvements in mfERG were detected in ring 1, 2 and 3, but not in the other rings. This may be explained by the fact that the photoreceptors in the 4th and 5th rings may have undergone apoptosis and the photoreceptors in the first 3 rings were found to remain in the dormant phase. Photoreceptor loss in the midperiphery in RP leads to hypertrophy and ectopic synaptogenesis of Müller cells located in the center [70]. The paracrine effect of Müller cells may explain the long-term preservation of the central 19-degree visual field [71].

**Fig. 9:**
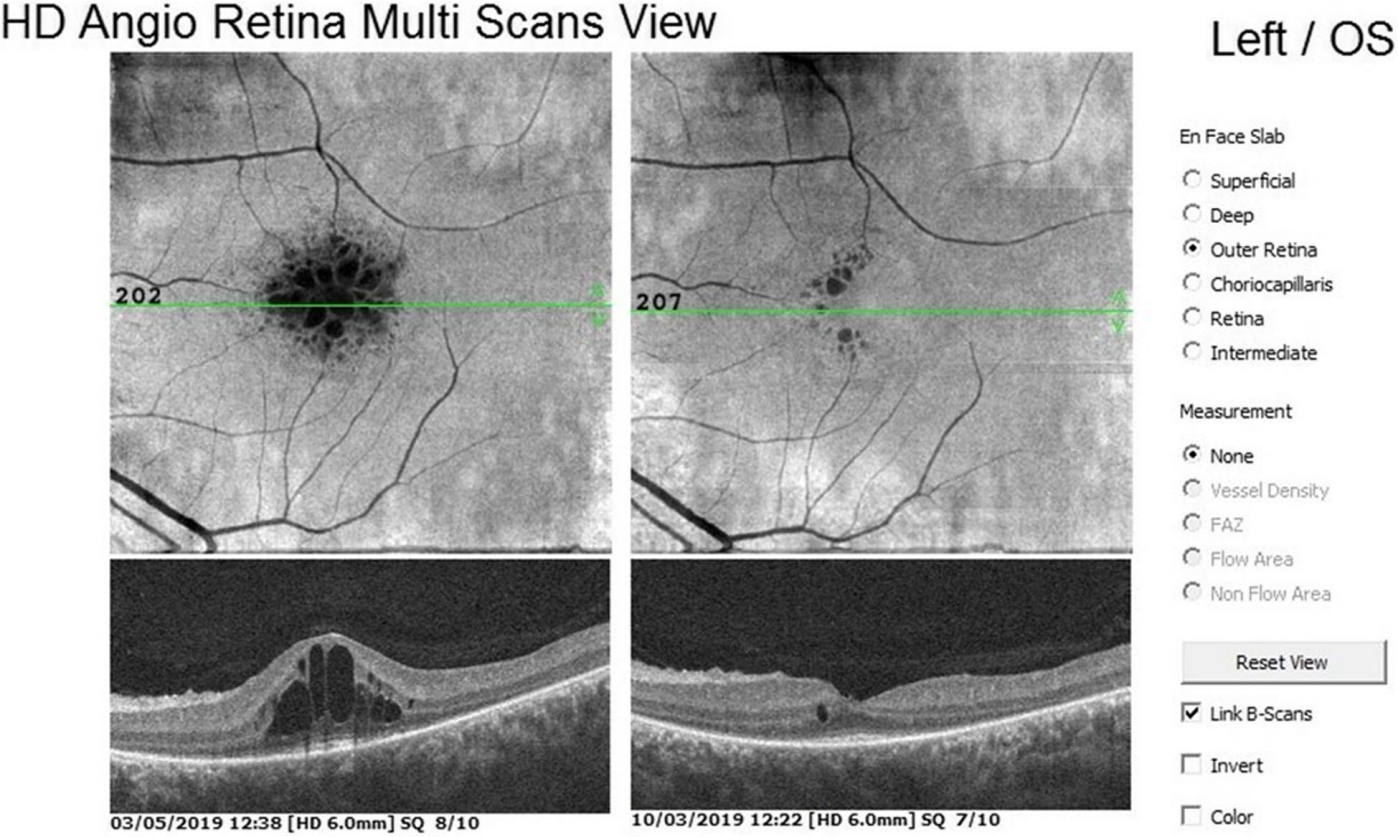
Regression of recalsitrant cyctoid macular edema after the WJ-MSC application (Table 1, patient no.16: left eye)

**Fig. 10:**
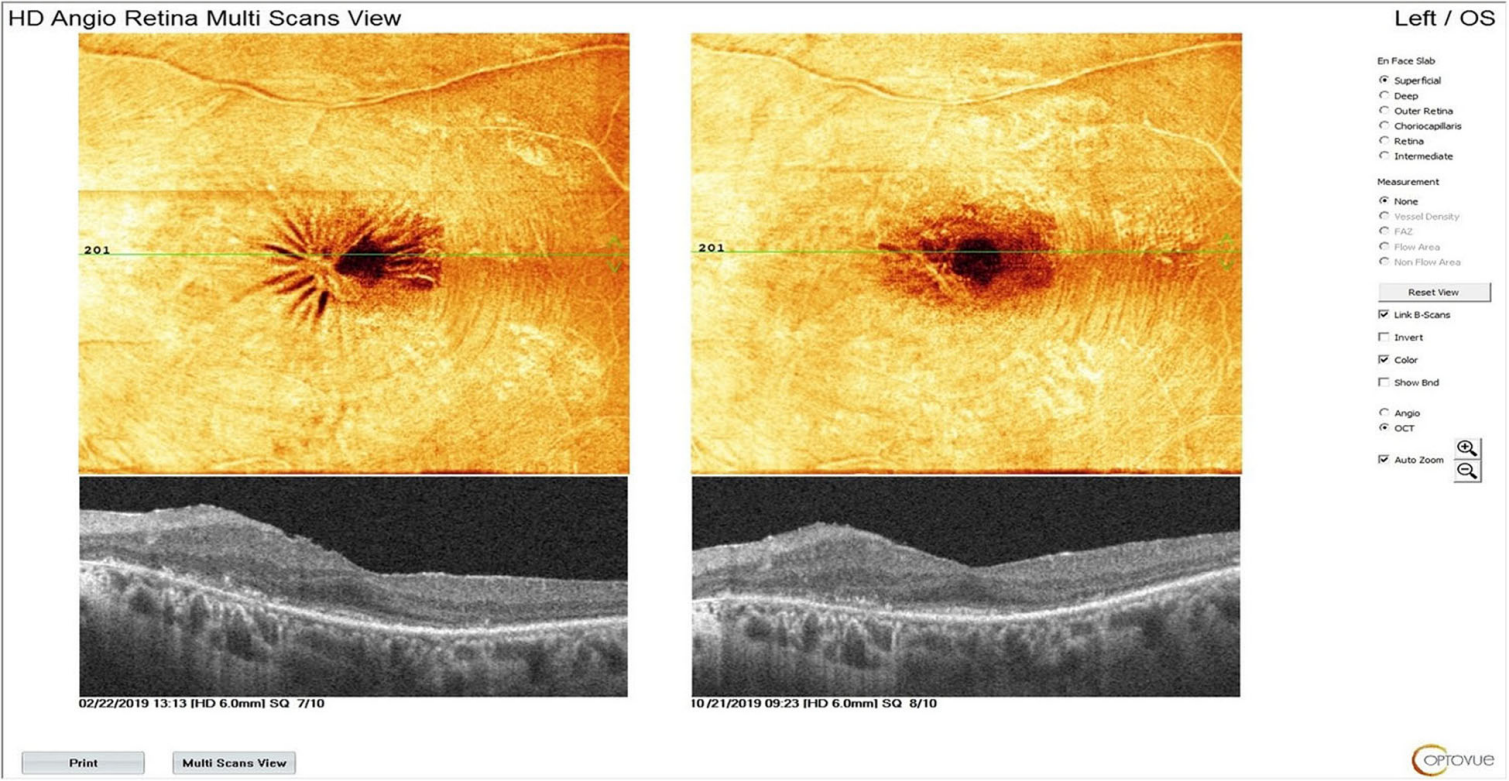
Releasing of epiretinal membrane contraction after the WJ-MSC application (Table 1, patient no.26: left eye)

**Fig. 11:**
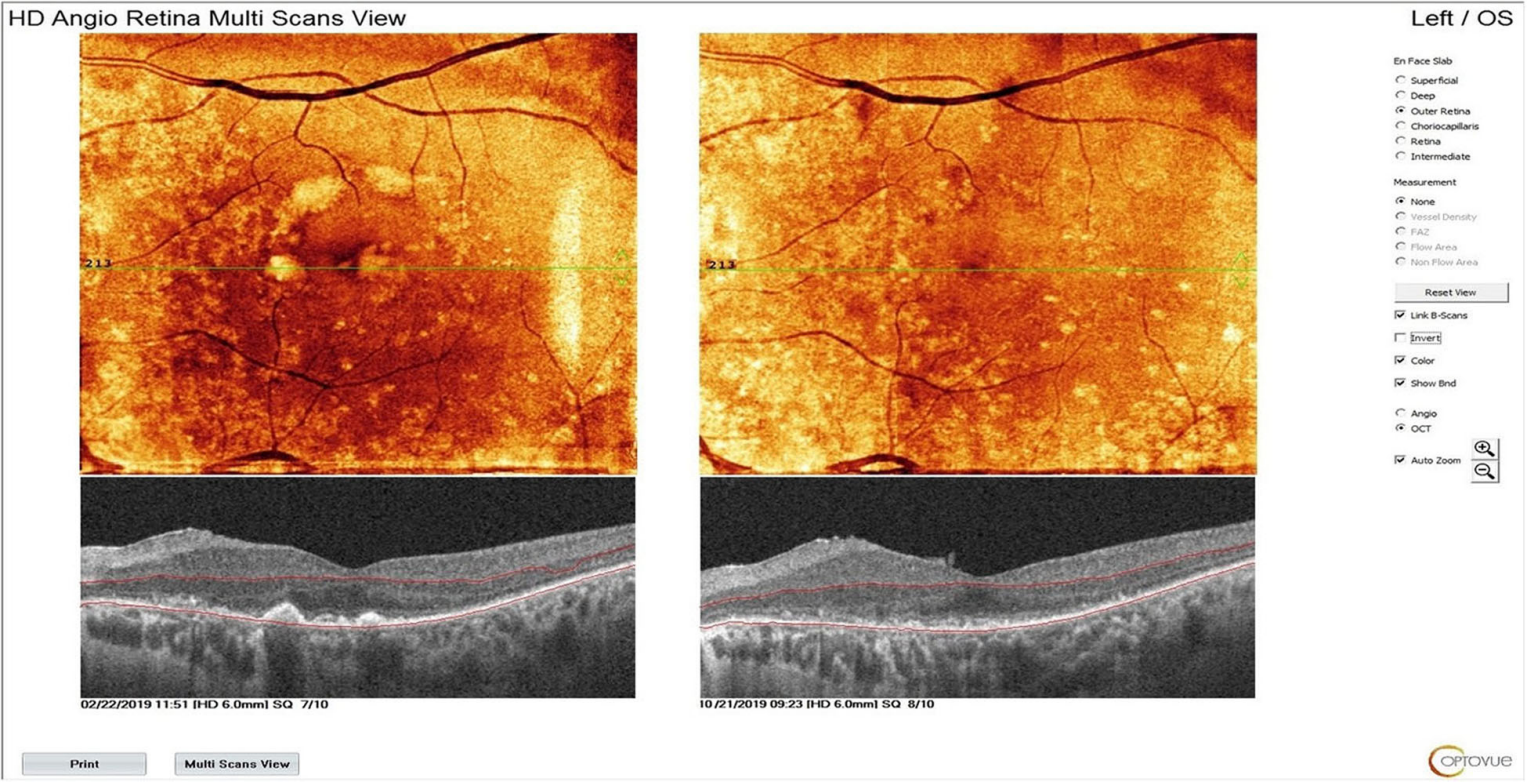
Dissapear of lipofuscin deposits after the WJ-MSC application (Table 1, patient no.26: left eye)

WJ-MSCs have a more rapid proliferation rate compared to adult tissue-derived MSCs because they are isolated from neonatal tissue; additionally, WJ-MSCs have decreased immunogenicity because they do not express MHC class II, CD40, CD80 and CD86 [20, 21]. WJ-MSCs also undergo less nuclear and mitochondrial mutations compared to MSCs collected from adult tissues. WJ-MSCs do not stimulate T-cell proliferation because they do not express MHC class II [45, 46].. WJ-MSCs have an effect on activated macrophages, reducing the secretion of inflammatory factors [12–21]. MSCs secrete factors that support cell survival and regulate adjacent cells in damaged tissue; hence, they can rescue the damaged naive nature of MSCs derived from an umbilical cord. In our study, we observed a decrease in intraretinal cystic edema, epiretinal membrane contraction and decreased lipofuscin volume (Figs. 9, 10 and 11). These conditions occured in the presence of chronic inflammation in RP. Chronic inflammation in RP progressively disrupts RPE functions and leads to Müller cell hypertrophy. Thus, macular edema in RP is resistant and will progress unless an immunomodulatory agent is used. We think that subtenon WJ-MSCs administration is effective for the suppression of chronic inflammation in the retina because of its immunomodulating properties. The decrease in lipofuscin volume can also be explained by the increase in RPE phagocytosis functions. Previous work has also found that many retinal disorders such as diabetic retinopathy, retinal vein occlusion and age-related macular degeneration have low-grade inflammation in their etiopathogenesis [27, 28, 56, 72].

In our previous clinical study, we used autologous platelet rich plasma (PRP) as a source of growth factors and demonstrated its efficacy in Turkish RP cases [5]. Growth factors obtained from PRP last for 4–6 months and require booster injections. Adipose MSCs can proliferate in tissues 5 times without karyotype changes [12, 45, 46] and WJ-MSCs can proliferate 25–300 times without karyotype changes [12–21, 45, 56]. This means that the paracrine-trophic effects of WJ-MSCs may persist for 3–7 years in the tissue where they are transplanted allogeneically. We believe that WJ-MSCs may be effective in patients who do not respond to PRP and that this may reduce the need for frequent injections.

The study has some limitations. First, the duration of action of WJ-MSC is unknown. Long-term research is necessary to determine at what intervals the WJ-MSCs application will be required. This is an important study limitation. Second, it is not known whether additional treatments such as electromagnetic iontophoresis or PRP injection may be useful to increase WJ-MSCs activity. Open label clinical research is advantageous for detecting pre- and post-treatment changes in homogeneous groups. The fact that one eye is the control group (without treatment) also supports the efficacy. The lack of placebo in the untreated eye is another limitation of the study in terms of understanding the effect of GFs that may be caused by surgical trauma. These limitations form the motivational basis for several future studies.

## Conclusion

RP is a genetic disorder that can cause blindness with outer retinal degeneration. There are few treatment options to slow or stop the progression of this disease. There is therapeutic potential in several novel approaches directed at limiting the course of photoreceptor loss, including growth factor injections (platelet rich plasma), gene therapy, and cell-based therapies. Regardless of the type of genetic mutation, administration of sub-tenon WJ-MSCs appears to be an effective and safe option. There are no reports of serious adverse events or ophthalmic/systemic side effects throughout 6 months of follow-up, although the long-term adverse effects are still unknown. As an extraocular approach, subtenon implantation of stem cells appears to be a reasonable way to avoid the devastating side effects of intravitreal/submacular injection. Further studies that include long-term follow-up are needed to determine the duration of efficacy.

## Data Availability

The datasets generated during and/or analyzed during the study are available from the corresponding author on reasonable request.

## Abbreviations

BCVA: Best corrected visual acuity
BDNF: Brain derived neurotrophic factor
bFGF: Basic fibroblast growth factor
cGMP: Current good manufacturing practise
CNTF: Ciliary neurotrophic factor
ERG: Electroretinography
ETDRS: Early treatment of diabetic retinopathy study
GFs: Growth factors
IGF: Insulin-like growth factor
MD: Mean deviation
mfERG: Multifocal electroretinography
MSCs: Mesenchimal stemcells
NGF: Neural growth factor
OCTA: Optical coherence tomography angiography
ORT: Outer retinal thickness
PRP: Platelet rich plasma
RP: Retinitis pigmentosa
RPE: Retinal pigment epithelium
Trk: Tyrosine kinase
VF: Visual field
WJ-MSC: Wharton’s jelly derived mesenchimal stem cell
